# Traumatic Brain Injury and Stem Cells: An Overview of Clinical Trials, the Current Treatments and Future Therapeutic Approaches

**DOI:** 10.3390/medicina56030137

**Published:** 2020-03-19

**Authors:** Giovanni Schepici, Serena Silvestro, Placido Bramanti, Emanuela Mazzon

**Affiliations:** IRCCS Centro Neurolesi “Bonino-Pulejo”, Via Provinciale Palermo, Contrada Casazza, 98124 Messina, Italy; giovanni.schepici@irccsme.it (G.S.); serena.silvestro@irccsme.it (S.S.); placido.bramanti@irccsme.it (P.B.)

**Keywords:** traumatic brain injury, stem cells, inflammation, clinical studies, stem cell transplantation

## Abstract

Traumatic brain injury represents physical damage to the brain tissue that induces transitory or permanent neurological disabilities. The traumatic injury activates an important inflammatory response, followed by a cascade of events that lead to neuronal loss and further brain damage. Maintaining proper ventilation, a normal level of oxygenation, and adequate blood pressure are the main therapeutic strategies performed after injury. Surgery is often necessary for patients with more serious injuries. However, to date, there are no therapies that completely resolve the brain damage suffered following the trauma. Stem cells, due to their capacity to differentiate into neuronal cells and through releasing neurotrophic factors, seem to be a valid strategy to use in the treatment of traumatic brain injury. The purpose of this review is to provide an overview of clinical trials, aimed to evaluate the use of stem cell-based therapy in traumatic brain injury. These studies aim to assess the safety and efficacy of stem cells in this disease. The results available so far are few; therefore, future studies need in order to evaluate the safety and efficacy of stem cell transplantation in traumatic brain injury.

## 1. Introduction

Traumatic brain injury (TBI) is the main cause of death in young people living in industrialized countries [1]. TBI is a condition caused by an external mechanical force responsible for brain dysfunction. This disorder can lead to temporary damage or permanent dysfunctions that induce severe cognitive, physical, and emotional disturbances [2,3]. Early intervention is important to minimize the development of brain injuries that could aggravate the patient’s condition. Surgery is often necessary for patients with more serious injuries to monitor and treat high intracranial pressure (ICP), decompress the brain if intracranial pressure has increased, or remove intracranial hematomas [4]. The management of ICP is also defined as life-saving treatment, as unstable ICP can compromise the patient’s life. The ICP management is performed by extraventricular drainage, as well as by intraparenchymal catheter or combined catheter [5]. The use of hyperosmolar substances such as mannitol and hypertonic saline solution represents a mild treatment route, currently used to reduce elevated ICP [6]. Other practices for managing ICP can be bed elevation, hyperventilation, and barbiturate coma.

The severity of the injury is clinically identified by the Glasgow Coma Scale (GCS). The GCS is a neurological rating scale used to assess patient’s level of consciousness after a head injury. This scale allows evaluating visual, verbal, and motor functions. The scoring system is based on three types of response to stimuli: best eye-opening (maximum 4 points), appropriate and consistent verbal response (maximum 5 points), and best motor response (maximum 6 points). The sum of these scores gives a score between 3 and 15 [7]. According to GCS, TBI is classified as mild to score of 13–15, moderate to a score of 9–12 and severe to a score of 3–8 [8].

The TBI is characterized by a primary lesion directly related to the traumatic event that causes impaired brain function. Within a few hours of the traumatic event, a complex cascade of cellular and molecular events that characterize the secondary lesion is triggered, responsible for further death/necrosis of brain tissue [9].

Today, pharmacological treatments of TBI is somewhat complex. Researchers are still studying new therapeutic strategies in order to reduce neurological damage [10]. Recently, the researchers have focused their studies on the use of stem cells (SCs) as a potential therapeutic option for the treatment of TBI. Hence, considering the neuroprotective role of SCs both in tissue reconstruction and in the ability to prevent cell death, they may be useful therapeutic strategies for the treatment of TBI [11]. In particular, neuronal stem cells (NSCs) and mesenchymal stem cells (MSCs)—such as bone marrow MCSs (BM-MSCs), adipose tissue-derived MSCs (AD-MSCs), and umbilical cord MSCs (UC-MSCs)—seem promising as new treatments for TBI [12].

This review aims to provide an overview of the effectiveness of stem cells in the management of TBI. For this purpose, we have collected the clinical trials and the studies approved by Local Ethics Committees that use stem cell transplantation as a therapeutic treatment in patients with TBI.

## 2. Traumatic Brain Injury

TBI is defined as an impairment of brain function caused by mechanical damage. It represents one of the main causes of death and disability in individuals aged between 1 and 45 years. The most common causes of TBI include falls, car accidents, assaults, and sports-related injuries [13]. According to recent incidence data, there is a higher peak among subjects in the adolescence, while a second peak is observed in the elderly. Furthermore, the incidence rates by gender show that males are at least twice more frequently affected than females [14].

The severity of the injury is commonly classified using the GCS score. TBI is defined as mild when patients retain good neurologic function and sequelae can be resolved completely without any treatment. Conversely, moderate or severe TBI can be fatal or cause severe disability which makes the quality of life negligible. Moreover, the injuries are typically classified as open or closed injuries. The open injuries involve a fracture of the cranial theca caused by a foreign object. While the closed injuries are marked by brain damage due to indirect impact without the entry of any foreign object into the brain. Most of the TBIs observed are of the closed type; however, a small percentage is represented by open TBI, generally experienced by gunshot injuries [15].

The TBI is characterized by two phases. The primary phase occurs at the time of exposure to external force and causes a mechanical breakdown of brain tissue. While the secondary phase has its onset hours after the traumatic event and represents the main cause of the worsening in the evolution of the TBI [10,16]. In TBI the self-regulation of blood flow is lost, this determines the increase in ICP with a consequent reduction in cerebral perfusion pressure and cerebral blood flow [17]. The break of normal cerebral blood flow occurs within 24 h of trauma and generally causes ischemic events responsible for the alteration of ionic gradients and consequent alteration of oxidative phosphorylation that leads to the accumulation of lactate. High levels of lactate cause acidosis responsible for the neuronal alteration, disruption of the blood–brain barrier and cerebral edema [18]. This can lead to the depolarization of neurons and the excessive release of excitatory neurotransmitters such as glutamate and aspartate, which induce an alteration in the homeostasis of calcium (Ca^2+^), potassium and sodium [19]. The increase in intracellular Ca^2+^ is responsible for activating a series of enzymes such as caspases and calpases that lead to cell death directly or indirectly through the activation of the apoptotic process. Additionally, an increase in the flow of Ca^2+^ in the mitochondria is also responsible for the production of reactive oxygen species (ROS) [20]. Oxidative stress also plays an important role in the pathogenesis of TBI damage. Indeed, the production of ROS has been shown to increase the neurodegeneration process that leads to lipid peroxidation of cell membranes, particularly at the axonal level [19,21,22]. Moreover, these events lead to the triggering of the inflammatory response characterized by macrophages, T lymphocytes, neutrophils, and glial cells that release pro-inflammatory cytokines as tumor necrosis factor-alpha (TNFα), interleukin-1beta (IL-1β), and interleukin-6 (IL-6) [16].

Therefore, the pathophysiology of TBI is complex, thus management, regardless of gravity, represents a great challenge. The treatment tools currently available for TBI are still not effective, with the exception of some therapies that are limited only to the reduction of secondary symptoms. Therefore, new therapeutic approaches are needed for the treatment of TBI.

## 3. Stem Cells and Traumatic Brain Injury

Currently, interventions to improve the lives of people with TBI in use—including drug treatments, surgeries, and rehabilitation therapy—provide poor outcomes [11]. The use of SCs in the treatment of TBI has recently entered the clinic as a possible therapeutic application [23,24]. SCs therapy is used in regenerative medicine to restore damaged neurons. SCs are cells that showed the multipotent capacity to differentiate toward different cell types and possess the capacity to renew themselves [25]. In different preclinical studies, conducted on TBI animal models, stem cell transplantation has promoted the improvement of several neurological parameters [12]. Specifically, NSCs and MSCs—such as BM-MSCs, AD-MSCs, and UC-MSCs—appear capable of regenerating damaged nerve tissue, as demonstrated in several in vivo studies, using animal models of TBI.

NSCs derived from the lateral ventricle, the dentate gyrus of the hippocampus [26]. Moreover, NSCs are a type of cell capable of self-renewal, they can differentiate into neurons, oligodendrocytes, and astrocytes. Additionally, NSCs are able to release cytokines and neurotrophic factors such as brain-derived neurotrophic factor (BDNF), glial cell-derived neurotrophic factor (GDNF), and insulin growth-factors-1 (IGF-1). Several studies suggest that transplanted NSCs can potentially repair and integrate neurons and glial cells at the injury site, due to their ability to release crucial molecules to maintain structural and functional plasticity [27]. NSCs, when administered in an experimental model of TBI, may be an effective, long-term treatment for neurological recovery after brain injury [28].

The MSCs are multipotent stromal cells that can be easily harvested from a variety of tissue sources such as bone marrow, umbilical cord, adipose tissue, placenta, and oral cavity [29]. Also, MSCs are potentially capable of differentiating into a variety of cell types including osteogenic, adipogenic, chondrogenic, and neural lineages [30]. Moreover, MSCs according to the definition of MSCs of must be positive for CD73, CD90, and CD105 surface markers. On the contrary, MSCs must be negative for CD45, CD34, CD14, CD11b, CD79a, and CD19 and for the major histocompatibility complex class II surface molecules [31]. MSCs promote the regeneration of damaged tissues through reducing inflammation response, recruiting local progenitor cells to replace lost cells and releasing trophic factors including BDNF, GDNF, vascular endothelial growth factors (VEGF), and nerve growth factor (NGF) [32]. Therefore, thanks to their proprieties, MSCs therapy can potentially be useful to repair damage following TBI [33]. Indeed, it was demonstrated that MSCs are able to electively migrate to the lesioned nervous tissue of the TBI rat and following differentiate towards neurons and astrocytes, in order to repair the damaged area, with consequent enhancement the motor activity after TBI [34].

The BM-MSCs derive from the bone marrow and showed the ability to differentiate into several cell lineage including neurons and glial cells [35]. It was demonstrated that BM-MSCs transplantation promotes neuroplasticity and neuronal regeneration through several mechanisms [36]. BM-MSCs showed anti-inflammatory effects by promoting the differentiation of lymphocytes. Additionally, BM-MSCs are able to release growth factors such as VEGF, BDNF, GDNF, NGF, epidermal growth factor (EGF), fibroblast growth factor (FGF), and neurotrophin-3 (NT-3), in this way leading on the repair of the lesioned area [37]. Several preclinical studies showed that BM-MSCs release trophic factors into the damaged sites, that inhibit apoptosis, promote angiogenesis and stimulate host progenitor cells to differentiate toward neurons and astrocytes. In this way, BM-MSCs showed the ability to repair the lesioned tissue and recovered function in animal models of TBI [38,39].

The UC-MSCs are obtained easily by treating the umbilical cord or the cord blood. The UM-MSCs compared to other stem cells showed several advantages such as a low immunogenicity power, less risk of rejection after transplantation, easy harvesting, and no ethical controversy [40]. These cells secreted neutrophil activator, NT-3, BDNF, VEGF, and FGF in order to induce neuronal regeneration and neuronal vascularization in the damaged area. It was demonstrated that UM-MSCs confer trophic support in the injured, induce the microglia/macrophage to remodel of the brain, leading to significant improvement of neurological functions [41].

The AD-MSCs are easily isolated from adipose tissue [42]. These cells have the ability to differentiate into cell types including neurons, endothelial-derived cells, and Schwann cells [43]. Additionally, AD-MSCs exhibit immunomodulatory properties thanks to the release of several cytokines including IL-10 and transforming growth factor-beta (TGF-β) [44]. Likewise, thanks also to the release of neurotrophic factors such as BDNF and GDNF, they showed neuroprotective properties [45]. It was demonstrated the AD-MSCs transplantation improvement motor activity in an animal model of TBI, suggesting that these cells might be considered for patients with TBI [46].

To date, studies report that the principally used stem cell routes of administration are the intravenous route and local administration by stereotactic injections [47]. The intravenous route has the advantage of being a non-invasive strategy, but it shows the limit that the percentage of infused cells that reaches the lesion site is very small. In addition, intravenous administration of stem cells implicates that these cells are retained by the pulmonary capillaries, thus compromising their therapeutic potential [48]. Hence, this route of administration results not very effective due to the large dispersion of cells between the different organs of the body, which requires the administration of a large number of stem cells. Compared to intravenous administration, local infusion of stem cells by stereotaxic injections is invasive but allows to inject stem cells directly into the injury site [49]. Therefore, this route of administration shows the advantage of reducing the number of stem cells used. However, the best route of administration has not yet been established, as each route shows its advantages and disadvantages.

## 4. Role of Stem Cell in Traumatic Brain Injury: Clinical Studies

In recent decades, several studies have shown the use of SCs as potential therapies in the treatment of neurological diseases such as TBI. This review provides an overview of the studies recorded on http://clinicaltrial.gov and the clinical trials approved by Local Ethics Committees.

In clinicaltrial.gov we carried out the research using the terms “head injury” and “stem cells”. This research led us to find 18 clinical studies. We have considered only clinical trials involving the use of stem cells as a therapeutic treatment for patients with TBI. We excluded clinical studies involving the use of drugs such as erythropoietin that stimulate the production of stem cells or other therapeutic interventions and clinical studies that involved the use of stem cells in diseases other than TBI. Additionally, on PubMed, we looked for studies approved by local ethics committees that evaluated the use of stem cells as a therapeutic treatment in patients with TBI. The keywords used for this research were “head injury”, “stem cells”, “clinical trial”, and “human”.

### 4.1. Clinical Trials Recorded in Clinicaltrial Gov

All the clinical studies (phases 1, 2, and 3) reported below have evaluated the safety and/or efficacy and tolerability of the administration from SCs in the TBI (Table 1). Most of these trials do not yet have available results as they are still in the patient recruitment phase.

A completed phase 1 clinical trial NCT00254722 was performed on 10 patients (aged 5–14 years) with acute TBI (within 24 h) diagnosed by GCS between 5 and 8. The aim of the study was to evaluate both the safety and efficacy of autologous bone marrow progenitor cell (BMPCs) transplantation in children with isolated TBI. The bone marrow samples were collected between 12- and 30-h post-injury at a dose of 3 mL/kg of body weight. The transplantation was performed by a single intravenous infusion of BMPCs at a dose by 6 × 10^6^ mononuclear cells/kg body weight in the 36 h after the lesions. The safety of the study was determined by monitoring cerebral and systemic hemodynamic both in the collection and transplant phase. Moreover, neurological events—such as seizures, alterations in GCS, stroke, as well as secondary organ injury—in the 12 h following the transplantation and up to 21 days after treatment were evaluated, in order to assess the efficacy of treatment. The results have not yet been posted.

The double-blind, controlled phase 2 study NCT02416492 has enrolled 61 male and female subjects (aged 18 years to 75 years) with chronic motor deficits secondary to TBI. The aim of the study was to evaluate the effectiveness, safety as well as tolerability in the stereotactic intracranial implantation of allogeneic modified bone marrow-derived mesenchymal stem cells (SB623). The SB623 cells are adult bone marrow cells that are transfected transitory way with a plasmid construct encoding the intracellular domain of human Notch-1. The participants were randomly divided into four groups: patients that had not received the surgical intervention (control group), and patients that received 2.5 × 10^6^, 5 × 10^6^, or 10 × 10^6^ SB623 cells. The SB623 were transplanted thought stereotactic intracranial injection. The primary outcome was evaluating the motor functions by using the Fugl–Meyer Motor Scale (FMMS) from baseline to 6 months in SB623 patients compared to the control group. Besides, it was assessed both changes in the Disability Rating Scale (DRS) and gait velocity, as well as an upper limb function from baseline at 6 months. The results of the study showed a significant improvement in the motor function by changes in FMMS at week 24 in patients treated with SB623 versus the control group. Besides, five severe side events were reported in patients treated with SB623 and three in patients in the control group. In contrast, there were no differences in the rate of side events occurred during the treatment in all experimental groups. Finally, the results of this study showed that intracranial SB623 transplantation was safe and effective, thanks to motor function improvements observed 24 weeks after treatment [50].

The cohort study NCT01575470 of 1/2 phase has enrolled 25 male and female patients (aged 18 years to 55 years) with acute serious TBI (GCS score 5–8) that showed injury at least 24 h before study start. This study was authorized by the University of Texas Health Sciences Center at Houston Committee for the Protection of Human Subjects and by the Memorial Hermann Office of Research. The goal of this trial was to assess the safety, feasibility, and efficacy of bone marrow mononuclear cells (BMMNCs) transplantation at the escalated doses. After 36 h of injury, from each patient were collected 3 to kg of bone marrow from anterior iliac crests. Before transplantation BMMNCs were tested for quality control including cell viability, nucleated cell count, gram stain, aerobic and anaerobic bacterial and fungal cultures, endotoxin and mycoplasma presence, colony-forming unit test, and multi-parameter flow cytometry analysis for cells characterization. The final doses of 6 × 10^6^ cells/mL/kg, 9 × 10^6^ cells/mL/kg, or 12 × 10^6^ cells/mL/kg in 0.9% saline containing 5% of human serum albumin were infused by intravenous injections. Twenty-five participants were assigned in 5 arms: 10 patients received no interventional surgical, 5 were included in control group 1 and 5 in control group 2; this additionally control group was included in order to include participants across the time spectrum of the trials. Five patients received 6 × 10^6^ cells/kg (low dose group), 5 patients received 9 × 10^6^ cells/kg (medium-dose group) and 5 subjects received 12 × 10^6^ cells/kg (high dose group). Neurological events such as seizures, changes in GCS score, cerebral vascular accident (CVA) were assessed both 12 h and 21 days after the infusion. Moreover, were assessed infectious morbidity up to 21 days after infusion and also the global functional status to assess both functional status and overall functioning in the 6 months post-injury. Additionally, inflammatory cytokines were measured in the plasma at baseline and 1 and 6 months after treatment. None of the patients manifested severe side events after collected bone marrow or after BMMNCs transplantation. After cell therapy, no serious pulmonary toxicity occurred in patients treated with a high dose of cells, but these events were not clinically significant. The cell therapy showed structural preservation of critical regions of interest brain tissue. The cytokine biomarker data showed a dose-dependent downregulation of the pro-inflammatory. Specifically, the IL-1β, interferon-γ (IFN-γ), and tumor necrosis factor-α (TNF-α) pathway show a statistically significant decrease in concentrations compared to the baseline. These results demonstrated that cell therapy, reduce the inflammatory response to injury. Finally, the autologous BMMNCs transplantation in patients with serious TBI is safe and feasible. This BMMNCs administration showed its efficacy in terms of maintenance of critical regions of central nervous tissue, probably due to a reduction of pro-inflammatory signaling [51].

The phase 2 interventional study NCT02525432 expected to recruit 55 male and female patients (aged 18–55 years) with acute TBI diagnosed by GCS between 5 and 8. The aim of this trial will evaluate the efficacy of intravenous BMMNCs transplantation. The patients will be divided into two groups; 33 subjects will be treated with autologous intravenous transplantation of BMMNCs within 48 post-TBI. The transplantation will be started with the lowest dose of 6 × 10^6^ cells/kg body weight followed by a higher dose of 9 × 10^6^ cells/kg of body weight. In contrast, 22 control patients will be undergoing a fake collection of bone marrow, as well as treated with a placebo. The primary outcome is to evaluate the macrostructural and microstructural properties both of gray matter and white matter, as well as the integrity of the regions in the corpus callosum, following the BMMNC autologous transplantation. The subjects will be evaluated by using magnetic resonance imaging (MRI) within 7–10 days at baseline, as well as to 1 and 6 months after lesions. The secondary outcome is to assess whether BMMNCs transplantation improves both functional and neurocognitive deficits, as well as the neuroinflammatory response following TBI. The subjects will be undergoing an evaluation of both size and splenic blood flow, also will be evaluated both inflammatory cytokines and infusion-related toxicity, as well as long-term safety. The toxicity and any complications associated with the infusion will be monitored within 14 days post-transplantation. Besides, both the safety and the results regarding the transplant will be evaluated after 1, 6, and 12 months from the injury. The results of this trial (estimated final data collection in October 2020) will make it possible to clarify the efficacy of BMMNCs transplantation.

The non-randomized study NCT02795052 aims to recruit 300 male and female participants (aged over 18 years) with damage to the central or peripheral nervous system documented by least 6 months, including TBI. The aim of the study is to show if the autologous BM-MSCs transplantation by intravenous and intranasal administration will provide an improvement in neurological functions in patients with some neurological conditions. The participants will be divided into two experimental groups. Patients in group 1 will be treated with BM-MSCs intravenous route. While the participants in group 2 will receive BM-MSCs intravenous and intranasal (lower 1/3 of nasal passages) route. The primary outcome will be to evaluate daily activities at 3, 6, and 12 months after the administration. The secondary outcome aims to assess deficits of neurologic function. This study is still active and the results have not been published yet.

The multicenter, randomized, blinded, placebo-controlled study NCT01851083 of phase 1/2 aims to recruit 50 male and female patients (aged 5–17 years) with severe TBI diagnosed by GCS between 3 and 8. The purpose of the study is to evaluate the effects of intravenous infusion of autologous BMMNCs on nervous tissue, as well as to evaluate the efficacy of cell therapy in neurocognitive and neurological function in children with severe TBI. The patients will be divided into two groups. The experimental group will be subjected to the collection of bone marrow and administration of BMMNCs in a single intravenous dose of 6 × 10^6^ cells/kg or 10 × 10^6^ cells/kg weight in the 48 h after lesions. Participants in the placebo group will undergo a fictitious collection of bone marrow and, subsequently, a single intravenous infusion of 0.9% sodium chloride. The primary outcome will evaluate by diffusion tensor MRI the conservation of both white and gray matter in the groups of patients treated and untreated after the trauma. The secondary outcome will measure the conservation of both the white matter and the gray matter in the CNS, as well as the improvement of functional and neurocognitive deficits. Furthermore, will be assessed the safety in the 7 days after treatment. For this trial, no result is available today.

The single-arm non-randomized study NCT04063215 of phase 1/2 hopes to recruit 24 male and female patients (aged 18–55 years) with sub-acute or chronic TBI (GCS score> 2 and ≤ 6) at least of 6 months. The study aims to evaluate both the safety and the efficacy of autologous AD-MSCs transplantation; moreover, it will evaluate the neurocognitive and neurological function and neuroinflammatory response after treatment. In adult patients, 2 × 10^8^ of AD-MSCs will be administered three times for 6 weeks. Furthermore, each infusion will be performed 14 days from the other. The primary outcomes will be evaluated by blood tests at baseline, as well as to 6 months and one year after infusion. The secondary outcomes involve an assessment of the macro and microstructural properties of the brain by MRI at baseline and 6 months post-treatment. Moreover, several tests will be performed for both functional and neuropsychological assessments at baseline, after 6 months and 1 year post-transplantation. Finally, will be assessed the inflammatory cytokines by cytometry analysis and monitoring the changes at 6 months and one year from baseline. The data from this trial will be available soon, as the final data collection is expected by August 2020.

The randomized phase 1/2 study NCT02959294 aims to recruit 200 male and female participants (16–70 years of age) with mild TBI or concussion syndrome. The aim of the study will be to evaluate both the safety and efficacy of adipose-derived stromal vascular fraction (AD-cSVF) in patients with TBI and concussion syndrome. The patients enrolled in the intervention arm will undergo a microcannula harvest of AD-cSVF. The primary outcome referring to the measurement of side events at baseline and 6 months after treatment. Besides, will be assessed the clinical symptoms associated with TBI and concussion such as recurrent headaches, amnesia, behavioral change, cognitive impairment, and recurrent sleep disturbances. The secondary outcomes will be evaluated through Beck’s questionnaire, in order to evaluate depression conditions. Furthermore, will be evaluated attention deficit and brain changes by MRI at baseline, 3 years, and 5 years after treatment. The results expected from this study will allow observing if the administration of AD-cSVF will be efficacy and safe for the long-term.

The phase 1 study NCT02742857 will be recruiting 20 male and female patients (15 years to 65 years) which present brain death caused by TBI with axonal injury shown by MRI. The study was designed using different therapeutic interventions including MSCs transplantation, infusion of intra-thecal bioactive peptides, transcranial laser therapy, and median nerve stimulation. The aim of the study is to show the possibility of cell inversion in the case of brain death induced by TBI with diffuse axonal injury. The primary outcome will be documented by clinical examination or electroencephalogram in the 15 days’ frame time. The secondary outcome is to provide a cerebrospinal fluid analysis, as well as the count and microbial evaluation. Furthermore, by using the MRI examination, both the presence of aseptic or bacterial meningitis, as well as any changes will be shown. Also, will be assessed blood pressure, pulse rate, oxygen saturation, as well as changes in breathing in the 15-day time frame. To date, no superior information is yet available. Final data collection is expected by June 2022.

### 4.2. Clinical Trials Approved by Local Ethics Committees

In this section, clinical trials published in indexed journals have been described. Many of these studies report the results obtained on treatment with stem cell therapy and provide important efficacy and safety data.

The study conducted by S. Wang et al. [52] aimed to evaluate the efficacy and safety of UC-MSCs transplantation in subjects with TBI. This trial was approved by the Medical Ethics Committee of the General Hospital of the Chinese People’s Armed Police Forces (Registration no. ChiCTR-TNRC-11001528). Forty participants with TBI ascertained by magnetic resonance imaging and computed tomography were enrolled for the study. Twenty of the 40 patients were randomly assigned to the stem cell treatment group, while the other 20 subjects were assigned to the control group. Prior to enrollment in the study, 15 of the 20 patients in the interventional group and 13 of the 20 patients in the control group had undergone cranial bone flap decompression and intracranial hematoma reduction surgery. While 5 patients in the interventional group and 7 in the control group, instead, after the trauma, received conservative medical treatment. For transplantation, the UC-MSCs were used between steps 6 and 8. Before transplantation, in order to increase the safety of therapy, multiple tests were performed on UC-MSCs including, morphological evaluations, spectrofluorometric tests for the identification of cell surface markers, sterility testing, and analysis to rule out the presence of endotoxins. Patients in the stem cell group, received 2 mL of stem cell suspension that containing 1 × 10^7^ SCs, via lumbar puncture in the lumbar 3–4 or 4–5 intervertebral space. This surgical intervention was performed four times in 5–7 days. To evaluate the efficacy of the treatment, the motor function of the limbs, the sensation, the balance and the ability to live independently, were assessed at the baseline and after 6 months of treatment (using the Fugl–Meyer Assessment and Functional Independence Measure scores). Prior to treatment, the two groups of participants showed equivalent baseline scores. After six months of treatment compared to the control group, patients treated with UC-MSCs showed a statistically significant improvement in motor function, sensitivity, and balance. The transplants patients, compared to the control group, also showed an improvement in mobility, locomotion, communication and ability to live independently. Four patients 48 h after transplantation experienced low intracranial pressure reactions with symptoms such as mild dizziness and headache. These adverse events resolved after lying down in bed or after being treated with intravenous saline infusions. In addition, during transplantation, clinical values—such as body temperature, oxygen saturation, blood pressure, and respiratory and heart rate—were monitored in all patients. No obvious anomalies were found in any subject. In conclusion, the results of this study demonstrate that the transplantation of UC-MSCs can be considered safe and can significantly improve several neurological parameters.

The retrospective cohort study conducted by G.P. Liao et al. [53] aimed to understand if BMMNCs transplantation within 48 h after trauma-induced a reduction of treatment intensity against managing elevated intracranial pressure relative in pediatric patients with severe TBI. This trial was authorized by The University of Texas Health Sciences Center at Houston Committee for the Protection of Human Subjects and approved by the Children’s Memorial Hermann Office of Research. The 29 TBI patients (5–14 years) with Glasgow Coma Score 5–8 and with trauma occurring <24 h within enrollment were recruited in this study. Ten patients were assigned to a treatment group and 19 in the control group. The 3–5 mL/kg of body weight was collected from posterior or anterior iliac crests. The mononuclear cells obtained before transplantation were undergone flow cytometry analysis for cell characteristics and tested for the presence of bacterial, fungal, and mycoplasma in order to increase the safety of transplantation. The subjects in the treatment group received 6 × 106 autologous BMMNCs cells/kg body weight IV within 48 h of trauma. The primary outcome of this study evaluated the Pediatric Intensity Level of Therapy scale in order to quantify the treatment of elevated intracranial pressure. Secondary outcomes included the Pediatric Logistic Organ Dysfunction score and days of intracranial pressure monitoring as a surrogate for the length of neurointensive care. In the treatment group, after transplantation was observed a significant reduction in the Pediatric Intensity Level of Therapy score beginning at 24 h post-therapy compared to the control group. The same results were obtained also in the Pediatric Logistic Organ Dysfunction score following the first week of transplantation. These differences may be due to the therapeutic effect of cell therapy. In conclusion, the results of this study showed that IV autologous BMMNCs transplantation reduces therapy intensity to maintained intracranial pressure and the severity of organ damage. Additionally, these findings demonstrated that these cells are able to reduce the effects of inflammation in early post-traumatic TBI.

This interventional cohort study conducted by C. Tian et al. [54] aimed to evaluate the efficacy and safety of autologous BM-MSCs in patients with TBI. This study was authorized by the ethical committee of the Hospital and Health Bureau of City. In the trial, 97 subjects with serious TBI for at least 1 month were enrolled. Twenty-four of the 97 showed a vegetative condition; while 73 patients manifested motor activity dysfunction. About 100 mL of bone marrow were harvested from each patient from posterior iliac crests. Before transplantation, BM-MSCs were characterized by flow cytometry analysis. Finally, 1 × 10^6^ cells/mL were administered by lumbar puncture between the lumbar vertebrae L3/L4 or L4/L5. In order to evaluate the efficacy of transplantation, 40 days after surgical intervention, the participants were followed-up for persistent vegetative state assessment and motor function analysis. None of the participants experienced severe side events. After two days of transplantation, 5 subjects showed transient fever and 2 participants perceived a slight headache. After 14 days of BM-MSCs transplantation. Thirty-eight of 97 participants showed enhancement of the TBI condition. Eleven of 24 participants before transplantation showed a vegetative state after treatment manifested enhancements in consciousness. Twenty-seven of 73 patients with motor dysfunction showed an enhancement in motor activity. Moreover, the age of participants affected the finding of cell transplantation, demonstrated that the young subjects enhancements the TBI state more than older patients. In conclusion, this study demonstrated that BM-MSCs transplantation is safe and effective in the management of serious TBI; furthermore, the early intervention in the subacute phase of this disorder showed better outcomes.

## 5. Conclusions

TBI is a very complex disease. Nowadays, there are no effective treatments able to reduce the effects of the primary injury, but only therapies capable of blocking their progression. In recent decades, NSCs and MSCs (BM-MSCs, AT-MSCs and UC-MSCs), have demonstrated to be a useful tool that can reduce the effects of post-traumatic brain injury. In several of the clinical studies described, BM-MSCs administrated via intravenous and via lumbar puncture, shown improvement in damaged brain areas. The same results were obtained using the UC-MSCs transplantation; however, the number of studies is fewer. Therefore, the available results encourage the use of both SCs and therapies described as useful for the treatment of TBI. The awaited outcomes and future studies will be necessary to exploit the use of cell transplantation for management TBI.

## Figures and Tables

**Table 1:** Clinical trials of stem cell therapy in TBI (https://clinicaltrials.gov/). The table shows the efficacy and safety of stem cell therapy for the treatment of patients with TBI.

Study Title	Identifier	Phase	Target Enrollment	Ages	Condition	Primary Outcome	Results	References
Safety of Autologous Stem Cell Treatment for Traumatic Brain Injury in Children	NCT00254722	1	10	5 to 14 years	Patients with TBI within 24 h after lesion	It has been evaluated the safety of BMPCs autologous transplantation	-	-
A Double-Blind, Controlled Phase 2 Study of the Safety and Efficacy of Modified Stem Cells (SB623) in Patients with Chronic Motor Deficit From Traumatic Brain Injury (TBI)	NCT02416492	2	61	18 to 75 years	Patients with motor deficits from TBI at least 12 months	It has been assessed the motor functions in patients with chronic TBI after stereotactic intracranial implantation of allogeneic SB623	The study results showed a significant improvement in motor function after 24 weeks of intracranial administration of SB623 cells. Furthermore, 5 severe side events were reported in patients treated with SB623 and 3 in the control patients. By contrast, there were no differences in the rate of adverse events emerging from the treatment between the two groups of patient	[50]
Treatment of Severe Adult Traumatic Brain Injury Using Bone Marrow Mononuclear Cells	NCT01575470	1/2	25	18 to 55 years	Patients with severe TBI within 36 h after injury	It was shown the safety of BMMNCs autologous transplantation after TBI	None of the study participants have shown severe side events after collection as well as the administration of BMMNCs transplantation. Likewise, cell therapy has demonstrated the structural conservation of the critical regions of interest brain tissue. Moreover, the authors have reported a decrease in the inflammatory response, as well as a statistically significant reduction in IL-1β, IFN-γ, and TNF-α following autologous BMMNCs transplantation.	[51]
Treatment of Adult Severe Traumatic Brain Injury Using Autologous Bone Marrow Mononuclear Cells	NCT02525432	2	55	18 to 55 years	Patients with TBI within 24 h after injury	Will be evaluated both the macrostructural and microstructural properties of gray matter, white matter as well as the integrity of the regions in the corpus callosum	-	
Neurologic Bone Marrow Derived Stem Cell Treatment Study	NCT02795052	-	300	Over 18 years	Patients with damage to the central or peripheral nervous system	Will be assessed the efficacy of intravenous and intranasal administration of autologous BM-MSCs by measuring the Activities of Daily Living at 3,6 and 12 months following the procedure	-	-
A Phase 2 Multicenter Trial of Pediatric Autologous Bone Marrow Mononuclear Cells (BMMNCs) for Severe Traumatic Brain Injury (TBI)	NCT01851083	1/2	50	5 to 17 years	Patients with severe TBI within 24 h after injury	Will be evaluated by using diffusion tensor MRI the conservation of both white and grey matter in the groups of patients treated and untreated after the injury	-	-
A Clinical Trial to Determine the Safety and Efficacy of Hope Biosciences Autologous Mesenchymal Stem Cell Therapy for the Treatment of Traumatic Brain Injury and Hypoxic-Ischemic Encephalopathy	NCT04063215	1/2	24	18 to 55 years	Patients with neurological injury at least 6 months	Will be evaluated the safety of autologous AD-MSCs transplantation in brain regions associated with specific neurocognitive deficits of patients with both subacute and chronic neurological injury	-	-
Use of Adipose-Derived Cellular Stromal Vascular Fraction (AD-cSVF) Parenterally in Post-Concussion Injuries and Traumatic Brain Injuries (TBI)	NCT02959294	1/2	200	16 to 70 years	Patients with mild TBI or concussion syndrome at least 1 months	The safety of AD-cSVF in patients with TBI and concussion syndrome will be assessed by measuring the side events at baseline and 6 months. Moreover, it will be assessed the clinical symptoms associated with TBI and concussion such as recurrent headaches, amnesia, behavioral change, cognitive impairment, as well as sleep disturbances.	-	-
Non-Randomized, Open-Labeled, Interventional, Single Group, Proof of Concept Study with Multimodality Approach in Cases of Brain Death Due to Traumatic Brain Injury Having Diffuse Axonal Injury	NCT02742857	1	20	15 to 65 years	Patients who present brain death caused by TBI with axonal injury shown by MRI	Will be demonstrated the reversal of brain death documented by clinical examination or electroencephalogram in the 15 days’ frame time	-	-

BMPCs: bone marrow progenitor cells; SB623: modified bone marrow-derived mesenchymal stem cells; BMMNCs: bone marrow mononuclear cells; IL-1β: interleukin-1beta; IFN-γ: interferon-γ; TNF-α: tumor necrosis factor-α; BM-MSCs: bone marrow mesenchymal stem cells; MRI: magnetic resonance imaging; AD-MSCs: adipose tissue-derived MSCs; AD-cSVF: adipose-derived stromal vascular fraction.
